# Women satisfaction with breastfeeding care in maternity hospitals: a survey from Italy

**DOI:** 10.1186/s13052-026-02201-0

**Published:** 2026-02-16

**Authors:** Riccardo Davanzo, Emanuela Lanfranchi, Silvia Perugi, Giuseppe Giordano, Maria Lorella Giannì, Maria Elisabetta Baldassarre, Massimo Agosti

**Affiliations:** 1 Research Centre for Nutrition, University of Insubria, Varese, Italy; 2 AST Pesaro-Urbino. Ospedale S.M della Misericordia, Urbino, Italy; 3 NICU, AOU Careggi, Firenze, Italy; 4 NICU, Ospedali Riuniti Villa Sofia-Cervello, Palermo, Italy; 5 NICU, Fondazione IRCCS Cà Granda Ospedale Maggiore Policlinico di Milano, Milano, Italy; 6 Dipartimento di Scienze Cliniche e di Comunità, Università degli Studi di Milano, Milano, Italy; 7 Department of Interdisciplinary Medicine‑Neonatology and NICU, University “Aldo Moro”, Bari, Italy; 8 Maternal and Child Department, Hospital “F. Del Ponte”, University of Insubria, Varese, Italy

**Keywords:** Breastfeeding, Postpartum care, Women satisfaction, Survey, Questionnaire

## Abstract

**Objectives:**

This study examines the satisfaction of women with the breastfeeding support they received in a sample of Italian maternity hospitals as part of postnatal care.

**Methods:**

Between November 15th and December 15th, 2023, a 20-items questionnaire was administered to 20 mothers who were consecutively discharged from each of the 26 Maternity Hospitals, that participate in a national project on the promotion of breastfeeding.

**Results:**

A total of 520 questionnaires were collected. Overall, the evaluation provided by women regarding the postnatal care was positive. The health team was perceived as welcoming, communicating clear information, practically and emotionally supporting, helpful in teaching how to take care of their baby and respectful of the mother-baby relationship. Moreover, the team was considered attentive to preventing and treating pain experienced by new mothers. In 20.2% of cases, women reported that the information provided about support resources after hospital discharge was lacking.

**Conclusions:**

The current study indicates that most women view postpartum care and breastfeeding support positively. However, positive results in studies assessing women's satisfaction must be interpreted with caution, as the relationship between expectations and satisfaction is not always clear. Additionally, we need to carefully consider what women report on insufficient information provided at hospital discharge, which may impact subsequent support.

## Key notes

Support for breastfeeding is an essential component of the postpartum care provided to the mother-baby dyad and should be assessed in MHs

Positive evaluation of women’ satisfaction with postpartum care and breastfeeding should be interpreted with caution, while negative results should be considered carefully

In Italy, information to women on available breastfeeding support after hospital discharge is reported to be suboptimal.

## Introduction

Assessing women’s satisfaction of the breastfeeding support is an essential component of quality care [1], related to healthy outcome including a reduced risk of postpartum depression [2]. The World Health Organization emphasizes that there is still insufficient attention given to this aspect, which would allow women to feel safe and to have a positive experience of childbirth [3]. The well-known variables that most significantly impact maternal satisfaction are the duration of labor, the type of delivery, the presence of perineal trauma, the effective pain management, the hospitalization of the newborn, the continuity of care, the consistency of clinical management by hospital professionals and, lastly and significantly, the breastfeeding experience [3]. A new mother, even after a physiological birth of a healthy baby, may encounter a series of breastfeeding problems (e.g., difficulties with the baby latching onto the breast, nipple cracks, etc.), which require competent help from healthcare professionals. Such help is not always available [4, 5], and, when available, it is often burdened by contradictions and ambiguities [6]. In other words, it must be recognized that the level of self-efficacy in breastfeeding, its initiation [7] and success [8] are essential components of the maternity experience not only of childbirth, but also of the postpartum period.

In Italy, an inter-societal project for the promotion of breastfeeding, known as the Hospital Policy on Breastfeeding (HPB) Project, is currently underway, as a joint initiative of the Italian Scientific Societies involved in perinatal care (the Italian Society of Neonatology, the Italian Society of Pediatrics, the Italian Society of Pediatric Nutrition, the Italian Society of Obstetricians and Gynecologists, the Italian Association of Hospital Obstetricians & Gynecologists, the Italian Society of Neonatal Nurses and the Italian Society of Pediatric Nurses), together with the National Midwife Board and the National Nurse Board and with Vivere Onlus, the Italian Federation of NICU Parent Associations [9].

The primary objective of the HPB project is to increase the rate of exclusive breastfeeding at discharge from Italian Maternity Hospitals (MHs), which, according to a survey conducted by the Task Force on Breastfeeding of the Italian Ministry of Health, shows an extremely wide range, from 20% to 97% [10].

With these premises, the present study aimed to explore the women satisfaction of the breastfeeding support within the context of postnatal hospital care provided in the MHs that joined the HPB. We illustrate data collected at the beginning of the HPB Project (T1), that will allow comparison with data to be collected at the end of the HPB Project (T2).

## Materials and methods

Out of the 104 Italian MHs participating in the HBP Project, 39 used a 20-items questionnaire (“Your Opinion Matters”; Supplementary Material 1) developed from a pre-existing validated questionnaire elaborated at Careggi Hospital, Florence. These MHs are public, smaller local as well as university and tertiary care facilities. They provide different level of care to a number of childbirths ranging from around 500 up to more than 3,000 per year, per single center. A questionnaire was proposed by the National Working Group on HPB to explore mother satisfaction on postpartum care, during the days spent in the Nursery/Rooming-in after childbirth. The 20-items questionnaire submitted to mothers was aimed to collect information on the education received in the postnatal ward, the staff provision of support, the characteristics of the communication between mothers and staff. Only mothers of healthy normal weight term newborns were invited to participate, in accordance with the sample selection of the main HPB study [9]. This questionnaire was constructed in Italian and translated into 10 languages (Albanian, Arabic, Chinese, English, French, Hindi, Romanian, Russian, Spanish and Ukrainian) by translators and/or cultural mediators connected to the hospitals participating in the study. Written consent was obtained from key-informants prior to commencement of each interview (Supplementary Material 2). The questionnaire was administered between November 15 and December 15 2023 to mothers consecutively discharged from MHs (T1). Lack of comprehension of the aforementioned multi-language questionnaire was an exclusion criterium. The number of women excluded for language barrier in the participating centers has not been recorded. Data collected at T1 will be compared with data collected between 1 and 30 November 2024 (T2), after an intervention bundle to promote breastfeeding conducted in the MH. Questionnaires were filled out independently and anonymously by one or both parents on the day of discharge, and returned before going home. Each MH was asked to collect 20 questionnaires administered to consecutively discharged mothers.

A frequency analysis on data collected at hospital discharge at T1 and a statistical inference on selected items were performed by a Digital Specialist (iDea Group).

## Results

Among the 39 public MHs that implemented the questionnaire on user satisfaction, 26 (66.6%) provided and uploaded the responses from 20 questionnaires through the SurveyMonkey platform, totaling 520 questionnaires. These 26 MHs belong to 7 Regions or Autonomous Provinces: 3 from Emilia-Romagna (AOU Modena, Bologna-Ospedale Maggiore, Bentivoglio Hospital), 1 from South Tyrol (Bruneck Hospital), 6 from Veneto (Vicenza, Arzignano, Valdagno, Mestre, Portogruaro, AOUI Verona), 10 from Lombardy (Varese, Tradate, Cittiglio, Sesto S. Giovanni, Milan-Niguarda, Milan-Macedonio Melloni, Manerbio, Desenzano, Gavardo, Bergamo-Giovanni XXIII), 1 from Liguria (Villa Scassi-Sampierdarena), 3 from Piedmont (Ciriè-Caselle, Turin-Maria Vittoria, Turin-Martini), 1 from Marche (Urbino), and 1 from Campania (Naples-Cardarelli). In summary, 24/26 MHs are located in Northern Italy. Nine out of 26 were tertiary care hospitals with a NICU.

Parents did not always answer all the questions. The questionnaires were completed by the mother in 73.3% of cases (381/520), by the father in 4.6% of cases (24/520), and jointly by both parents in 21.9% of cases (114/520).

Women having an Italian cultural background were 68% of cases (354/520), a non-Italian cultural background 26.0% of cases (135/520) and a mixed cultural background 6.9% (31/520).

The length of stay for the mother-baby pair was less than 2 days in 3.1% of cases (16/520), 2–5 days in 86.5% of cases (450/520), and more than 5 days in another 10.0% cases (52/520). Women with a non-Italian cultural background have a hospital stay at childbirth more than 5 days more often than Italian women (23/354 vs. 24/134)(*p* = 0.0027).

Women gave an overall positive evaluation of postpartum care across many different aspects (Table 1). In most cases, parents felt supported by a welcoming and helpful team, that communicated information clearly, showed empathy and provided emotional support. The team was also attentive to preventing and treating eventual pain symptoms experienced by new mothers. Despite the recognized workload, the healthcare team showed respect for the mother-baby relationship and taught parents how to care for their babies. According to personal experience, women felt they could recommend the same hospital for future childbirths.

**Table 1:** Satisfaction with postpartum care and breastfeeding among 520 mothers in 26 Italian MHs

Items Explored by the user satisfaction questionnaire	Yes	Somewhat	Little/No	Total Responses
	*N* (%)	*N* (%)	*N* (%)	*N* (%)
1. Clarity of information about:				
a. Child and their tests/exams/visits	458 (88.2%)	59 (11.4%)	2 (0.4%)	519 (100%)
b. Breastfeeding and its issues	457 (87.9%)	57 (11.0%)	6 (1.1%)	520 (100%)
2. Consistency of breastfeeding information among different staff members	414 (80.7%)	79 (15.3%)	20 (4.0%)	513 (100%)
3. Provision of brochures/sheets/informative booklets about Community Services for seeking help, especially regarding breastfeeding, in case of need	369 (72.9%)	35 (6.9%)	102 (20.2%)	506 (100%)
4. Commitment to prevention and/or treatment of my pain (e.g., due to episiotomy, hemorrhoids, cesarean section wound, nipple irritation or cracks)	440 (86.3%)	59 (11.6%)	11 (2.1%)	510 (100%)
5. Staff awareness of my general situation and breastfeeding	464 (89.4%)	52 (10.0%)	3 (0.6%)	519 (100%)
6. Effective staff response to:				
a. My baby’s needs	469 (96.1%)	18 (3.7%)	1 (0.2%)	488 (100%)
b. My needs as a new mother	447 (91.0%)	42 (8.6%)	2 (0.4%)	491 (100%)
7. Adequate emotional support from the staff	452 (87.9%)	55 (10.7%)	7 (1.4%)	514 (100%)
8. I was helped to keep my baby close to me	469 (92.7%)	31 (6.1%)	6 (1.2%)	506 (100%)
9. The staff encouraged me during difficulties in managing my baby	452 (89.3%)	46 (9.1%)	8 (1.6%)	506 (100%)
10. The staff helped me to develop an emotional bond with my baby	412 (82.2%)	74 (14.8%)	15 (3.0%)	501 (100%)
11. The staff taught me how to take care of my baby	455 (89.0%)	45 (8.8%)	11 (2.2%)	511 (100%)
12. The staff always introduced themselves by name and function	358 (69.8%)	98 (19.1%)	57 (11.1%)	513 (100%)
13. I felt empathy from the staff	420 (86.6%)	55 (11.3%)	10 (2.1%)	485 (100%)
14. The team showed respect for my baby and me	471 (96.3%)	18 (3.7%)	0 (0%)	489 (100%)
15. The atmosphere among the staff was pleasant	439 (90.0%)	43 (8.8%)	6 (1.2%)	488 (100%)
16. We felt welcomed by the staff	473 (91.7%)	37 (7.1%)	6 (1.2%)	516 (100%)
17. The staff gave proper attention to my baby and me despite their workload	461 (89.2%)	50 (9.7%)	6 (1.1%)	517(100%)
18. My cultural background was considered	331 (80.9%)	20 (4.9%)	58 (14.2%)	409 (100%)
19. The staff was always available to listen to me	459 (92.9%)	33 (6.7%)	2 (0.4%)	494 (100%)
20. I would recommend this hospital to other future parents	475 (96.7%)	14 (2.9%)	2 (0.4%)	491 (100%)

**Fig. 1:**
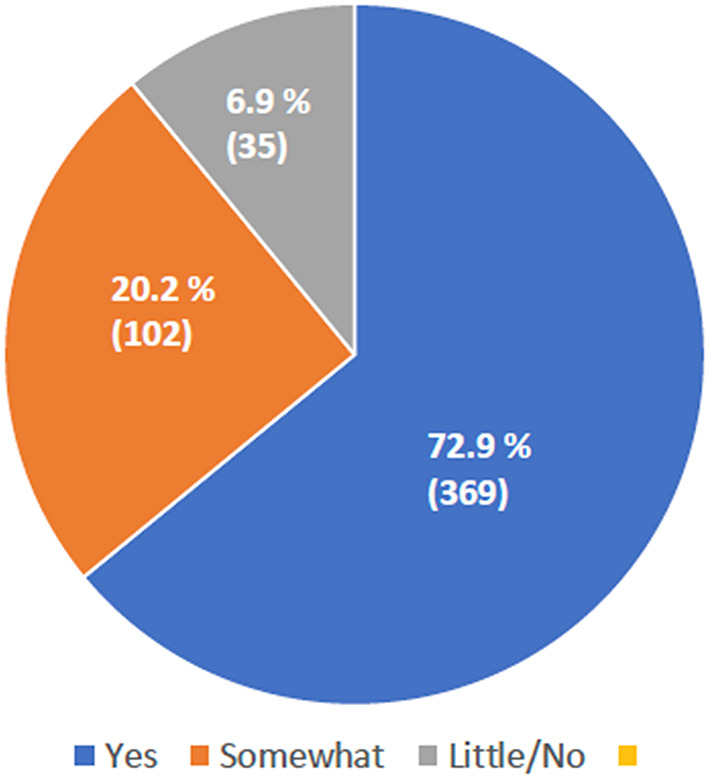
Provision of information by mothers/families for seeking support with breastfeeding after hospital discharge. Percentages and absolute numbers are reported

Nevertheless, some critical issues emerged from the survey:

1. 11.1% of families reported that the healthcare staff did not introduce themselves by name and role when interacting with them. This issue was equally reported by Italians (48/353) and by non-Italians (11/134)(*p* = 0.882).
2. In 14.2% of cases, the staff was deemed not to adequately consider the cultural background of women, although no differences were observed between Italian and non-Italian populations. *p* = 0.858)
3. Finally, in a relevant percentage of the collected questionnaires (20.2%), information provided to women about the available support resources after hospital discharge was reported as lacking (Fig. 1). No differences were observed between Italian and non-Italian populations (*p* = 0.792).

## Discussion

Patient satisfaction is increasingly recognized as a measure of quality in healthcare settings, including maternal childbirth care [11–14] and intensive neonatal care [15–17].

Care during childbirth and breastfeeding should be empathetic towards women, respectful of their choices, and create a relaxing and safe environment [2]. Encouraging an active participation of the woman in the decision-making process, an open communication and respect for the mother’s preferences contribute to a positive experience [2], though women’s expectations may sometimes be influenced by their knowledge and acceptance of the care practices at specific MH, whatever they may be [18].

Regarding breastfeeding, qualified support significantly influences overall satisfaction and the success of the breastfeeding experience. Institutions that encourage a family-centered care environment tend to receive more positive evaluations for childbirth and breastfeeding support [19].

Questionnaires designed to explore mothers’ satisfaction with their postnatal care primarily focus on some key aspects: checking the baby’s and the mother’s health, providing education on baby care and breastfeeding, offering emotional and practical support and addressing the mother’s questions or concerns.

In the current study the evaluation provided by women regarding the care they received was overall positive. However, there are several reasons why we must be cautious in interpreting positive results and avoid drawing hasty conclusions [20].

Although survey questionnaires are a popular tool to obtain information on patient satisfaction, it is recognized that their use implies intrinsic methodological problems. Particularly, it is not clearly known the nature of the relationship between expectations and expression of satisfaction [21]. Patients may have important beliefs which influence their expressions of satisfaction [22]. Satisfaction may depend on factors originating beyond the healthcare, that we want to evaluate. These factors may include individual socio-demographic characteristics [23], personal values, prior knowledge of the functioning and resolution capacity of a specific health facility, or even culturally determined expectations, which can vary and may be set at a low level.

On their part, women may be variously confident in the competence of the health staff, while are more critically focused on personnel behavior and communication, that consequently are expected to play a major influence on their overall opinion.

Generally, questionnaire surveys show a prevalence of positive results. In a very dated review of studies on patient satisfaction [24], the average percentage of satisfied patients was as high as 77.5%, suggesting an uncritical or possibly passive attitude on the part of interviewees. On the contrary and noticeably, patients show lower level of satisfaction, when the administered questionnaire on satisfaction allows to respond to more opened questions.

The validity of the questionnaires used to evaluate women’s satisfaction with childbirth and postnatal care is crucial. In fact, it ensures that the instrument effectively captures the concepts it is designed to evaluate and produces consistent results across different contexts and populations. Particularly, questionnaires should be periodically reviewed and updated to reflect the evolving needs and expectations of patients [25].

Unfortunately, the measure of satisfaction has no standardized scale [24, 26] and despite maximum efforts the process of validation still remains incorrigibly tricky, ultimately leading to a limited number of available validated questionnaires [27–29].

Having established all these premises, we should appreciate that any questionnaire survey inevitably maintains a certain degree of inconsistency and that consequently an expressed dissatisfaction of women deserves more consideration than satisfaction.

This study depicts a positive picture of the provision of postpartum hospital care. However, the results cannot be generalized to the entire country as derive from a sample that is selected in many respects. First, the MHs are mostly located in Northern Italy, a geographic area that generally has higher breastfeeding rates (geographical bias) [30]. This may not only be due to socio-economic and cultural differences between geographic areas, but also to possibly better breastfeeding promotion, protection, and support provided by healthcare facilities. Second, the 26 MHs participating in the HPB Project may be more motivated about breastfeeding and more aware of the importance of the mother-baby relationship, thus offering more appropriate care [9]. This introduces a potential sampling bias. Third, these 26 MHs, by choosing to participate in the survey, have demonstrated an interest in being evaluated by users. It is possible that these MHs are more interested to know the opinions of mothers or simply confident that the survey would not reveal negative results regarding breastfeeding support (selection bias). Fourth, filling out a satisfaction questionnaire is usually subjected to a moderately high non-response rate, that is another major bias to be accounted. The mean response rate in 210 studies on patient satisfaction published in 1994 in different health journals was reported 72.1% [31], but we have no precise information on the response rate of mothers in our study.

Fifth, a positive opinion by mothers might be also influenced by an acquiescence bias or by the indulgence toward their own experience and reluctance to express critical comments (the so-called “gratitude bias”).

Our study has ignored the person-related characteristics, that are well known potential confounders [23]. Actually, the principal goal of our study was not to make inference between the level of satisfaction and sub-groups of the sample categorized according to maternal age, education, employment, parity, type of delivery and newborn birth weight.

A major strength of the study lies in exploring satisfaction on breastfeeding support within the broader context of postpartum care in a sample of MHs that accounts to 14% of the 172 hospitals located in Northern Italy [32].

The results might demonstrate that, within this selected sample, there is a widespread positive cultural approach where the mother-baby relationship is central to hospital care, which is appreciated by women. However, a minority (19.9%) of women did not receive complete information upon discharge on how to orientate themselves, especially when seeking help with breastfeeding. According to UNICEF/WHO Baby Friendly Initiative, families returning home should receive written and verbal information on how to obtain competent and timely breastfeeding support, as milk production may not be fully established yet [33]. Proper discharge from the hospital involves not only ensuring that the mother’s caregiving skills are adequate, but also anticipating that she may encounter difficulties, particularly with breastfeeding, and may need competent support. This support should be provided by the same MH or, preferably, by health facilities in the community, or, complementarily, by lactation consultants and/or peer-to-peer support [34]. The family should receive an appointment for a follow-up visit within 24–72 h of returning home [35], but they should also have the information needed to obtain breastfeeding support if required. If in-person support is not possible, remote support via video call is effective, without overlooking the possibility of a simple traditional phone call [36, 37].

In the current era, unfortunately, professional frustrations are common, at least in Italy, among those who work daily in hospitals, given the progressively limited availability of human and material resources [38]. Therefore, the data for postnatal care satisfaction from this study might represent a justifiable reason of pride for a group, though selected, of MHs. At the same time, they could serve as an inspiration for other MHs, whether or not they participate in a breastfeeding promotion project such as the HPB Project.

Finally, as satisfaction surveys may orientate to shape maternity care policy and its organization, we should be aware that an over emphasis on positive outcomes actually implies the risk to promote the *status quo*, possibly ignoring further improvements [39].

In our study sample, 11.1% of women reported that healthcare staff do not introduce themselves by name and role when communicating with them, independently of the cultural background of the families. As postpartum healthcare team have the responsibility to ensure that the care provided is not just patient-oriented but also family-centered, it is important for them to properly address mothers/families. Appropriate communication behaviors are required not only as an elementary demonstration of courtesy, but also to build a positive perception of the quality of care among families [40, 41].

## Conclusions

The postpartum hospital care and breastfeeding support in a group of the MHs participating to a breastfeeding promotion project seem to be well-received by the great majority of women. However, the high level of satisfaction reported for postpartum care may not accurately reflect the true experiences of mothers. It is preferable to point the negative outcomes that actually identify area of potential improvement of postpartum care. According to our study, MHs staff are challenged to: (1) improve communication with women in terms of introducing themselves and better understanding the cultural background of families and, (2) provide comprehensive and routine information to mothers on breastfeeding support after hospital discharge, possibly review available support from local resources.

Ultimately, effective postnatal support to mothers/families in the rooming-in ward requires dedicated time and presence by health professionals having specialized breastfeeding and following integrated care protocols. This approach together with an information given to mothers/families at hospital discharge on how to search professional quality support in case of need with breastfeeding enhances maternal self-efficacy and may reduce the negative impact of post-discharge suboptimal advice.

## Data Availability

The data supporting the findings of this study are available from the corresponding author upon reasonable request. Any restrictions on data availability are due to ethical or legal considerations.

## Abbreviations

HPB: Hospital Policy on Breastfeeding
MH: Maternity Hospital
NICU: Neonatal Intensive Care Unit
